# Sexual health clinic attendance and non-attendance in Britain: findings from the third National Survey of Sexual Attitudes and Lifestyles (Natsal-3)

**DOI:** 10.1136/sextrans-2017-053193

**Published:** 2017-09-29

**Authors:** Clare Tanton, Rebecca S Geary, Soazig Clifton, Nigel Field, Katie L Heap, Fiona Mapp, Gwenda Hughes, Anne M Johnson, Jackie A Cassell, Pam Sonnenberg, Catherine H Mercer

**Affiliations:** 1 Institute for Global Health, University College London, London, UK; 2 Department of Social & Environmental Health Research, London School of Hygiene & Tropical Medicine, London, UK; 3 Department of HIV and STIs, National Infection Service, Public Health England, London, UK; 4 Division of Primary Care and Public Health, Brighton and Sussex Medical School, Brighton, UK

**Keywords:** sexual health clinic, attendance, sexual behaviour, probability survey, United Kingdom, surveys and questionnaires, unsafe sex, general practice, prevalence

## Abstract

**Objectives:**

In Britain, sexual health clinics (SHCs) are the most common location for STI diagnosis but many people with STI risk behaviours do not attend. We estimate prevalence of SHC attendance and how this varies by sociodemographic and behavioural factors (including unsafe sex) and describe hypothetical service preferences for those reporting unsafe sex.

**Methods:**

Complex survey analyses of data from Britain’s third National Survey of Sexual Attitudes and Lifestyles, a probability survey of 15 162 people aged 16–74 years, undertaken 2010–2012.

**Results:**

Overall, recent attendance (past year) was highest among those aged 16–24 years (16.6% men, 22.4% women), decreasing with age (<1.5% among those 45–74 years). Approximately 15% of sexually-active 16–74 year olds (n=1002 men; n=1253 women) reported ‘unsafe sex’ (condomless first sex with a new partner and/or ≥2 partners and no condom use, past year); >75% of these had not attended a SHC (past year). However, of non-attenders aged 16–44 years, 18.7% of men and 39.0% of women reported chlamydia testing (past year) with testing highest in women aged <25 years. Of those aged 16–44 years reporting unsafe sex, the majority who reported previous SHC attendance would seek STI care there, whereas the majority who had not would use general practice.

**Conclusion:**

While most reporting unsafe sex had not attended a SHC, many, particularly younger women, had tested for chlamydia suggesting engagement with sexual health services more broadly. Effective, diverse service provision is needed to engage those at-risk and ensure that they can attend services appropriate to their needs.

## Introduction

Over the past 30 years, substantial changes in sexual behaviour in Britain have been observed including a decline in the age at sexual debut, larger numbers of partners, a wider range of sexual repertoires and increases in the proportion of the population reporting same-sex sexual experiences.^1 2^ In addition, a number of strategies to improve sexual health and access to sexual healthcare have been implemented since 2000, as in other countries.^3–8^ All aim to reduce risk behaviour and to improve access to STI testing and treatment. In an attempt to achieve this, genitourinary medicine (GUM) clinics were modernised, 48 hours waiting time targets for GUM implemented and the role of primary care expanded, resulting in an expansion in the range and accessibility of STI services.^9^ National STI-specific intervention programmes, such as chlamydia screening, were also implemented.^10 11^

Using data from the British National Surveys of Sexual Attitudes and Lifestyles (Natsal), we have previously demonstrated substantial increases in sexual health clinic (SHC) attendance since 1990.^9 12^ Although large and increasing proportions of the population are accessing sexual health services and testing for STIs, many people at risk of STIs may not. For example, in Natsal-3 (2010–2012), two-thirds of participants in whose urine chlamydia was detected did not report having had a chlamydia test in the past year, and more than three-quarters had not attended a SHC during this time.^9^ Unlike surveillance data^13^ population-based probability surveys like Natsal can capture data on those who do not attend services, which are essential for informing the design and delivery of sexual health services and STI control programmes.

The aim of this study is to describe the characteristics of SHC attenders and non-attenders reporting unsafe sex in the past year, as an indicator of potential need for sexual health services and to explore hypothetical service preference in those attending and not attending SHCs. This expands on our previous work^9^ by extending the age range to 74 years and also restricting analyses to those reporting unsafe sex.

## Methods

### Participants and procedures

Full details of the methods used in Natsal-3, a household-based survey, have been reported elsewhere.^1 14^ Briefly, we used a multistage, clustered, stratified probability sample design. Postcode sectors throughout Britain were used as the primary sampling units (PSUs). Before selection, PSUs were stratified (by region, population density, the proportion of the population aged under 60 and the proportion of households with a head in a non-manual occupation) and were then selected systematically with a probability of selection proportional to the total number of addresses. Within each sampling unit, addresses were randomly selected and trained interviewers visited all sampled addresses, identified individuals in the eligible age range and randomly selected one individual to invite to participate. In total, 15 162 men and women aged 16–74 years resident in Britain were interviewed in English between September 2010 and August 2012 through a combination of face-to-face computer-assisted personal interviews and computer-assisted self-interview (CASI) for the more sensitive questions. Non-response to individual questions (missing data) was low (generally 1%–3%). Data were weighted to account for unequal selection probabilities in terms of age and the number of adults in the eligible age range at an address and differential non-response to correct for differences in sex, age and Government Office Region.^14–16^ The Natsal-3 study was approved by the Oxfordshire Research Ethics Committee A (reference: 09/H0604/27) and participants provided oral informed consent to take part.

### Measures

In the CASI, participants were asked ‘Have you ever attended a SHC (GUM clinic)?’ and, if yes: ‘When was that? (The last time if more than once)’. Response options were: *less than 1 year ago, between 1 and 5 years ago, between 5 and 10 years ago, more than 10 years ago.* Participants were also asked, ‘If you thought that you might have an infection that is transmitted by sex, where would you first go to seek diagnosis and/or treatment?’. Response options were:

*General practice (GP) surgery*

*Sexual health clinic (GUM clinic)*

*NHS Family planning clinic/contraceptive clinic/reproductive health clinic*

*NHS Antenatal clinic/midwife*

*Private non-NHS clinic or doctor*

*Pharmacy/chemist*

*Internet site offering treatment*

*Youth advisory clinic (eg, Brook clinic)*

*Hospital accident and emergency (A&E) department*

*Somewhere else*

‘Sexual health clinic (GUM clinic)’ was not defined for participants, and throughout this paper, we refer to this as ‘SHC’ for brevity.

In this analysis, participants were considered to have had ‘unsafe sex’ in the past year if they reported: not using a condom at first sex with a new (vaginal or anal sex) partner in the past year and/or two or more sexual partners in the past year and no condom use in that time. This measure is in line with national clinical recommendations for those who have had unprotected sex with a new partner to have a sexual health check^17^ and epidemiological data on behaviours associated with increased risk of STIs^9^ among those with multiple partners. Those who had only had oral sex partners in the past year were not classified as having had unsafe sex.

### Statistical analysis

We used Stata V.14.1 for complex survey analysis to incorporate weighting, clustering and stratification of the Natsal-3 data.^18^ We present prevalence estimates of SHC attendance (ever, in the past 5 years and in the past year) by sex (participants could only identify as male or female) and age group among the sexually experienced population, defined as those aged 16–74 years who reported at least one sexual partner ever. We then used logistic regression to calculate age-adjusted ORs to investigate how recent SHC attendance (past year) varied by key sociodemographic and sexual health and behaviour factors among all sexually active men and women (those reporting at least one sexual partner in the past year). We then focused our analysis on those reporting unsafe sex (past year) as a way of identifying individuals who may be at risk of STIs and who may therefore have a need to engage with sexual health services. Among this group, we used logistic regression to explore the factors associated with *non-attendance* at a SHC in the past year, in order to identify those less likely to attend. We present descriptive statistics of preferred location for STI diagnosis and/or treatment among 16–44 year olds classified as having had unsafe sex in the past year (in order to ensure that the hypothetical preferences reflected those who were more likely to require these services in the future), by sex and time since last SHC attendance. Data for this last analysis are presented for 16–44 year olds reflecting the age range asked this question.

## Results

### Prevalence of SHC attendance by age among sexually experienced men and women

**Figure 1:**
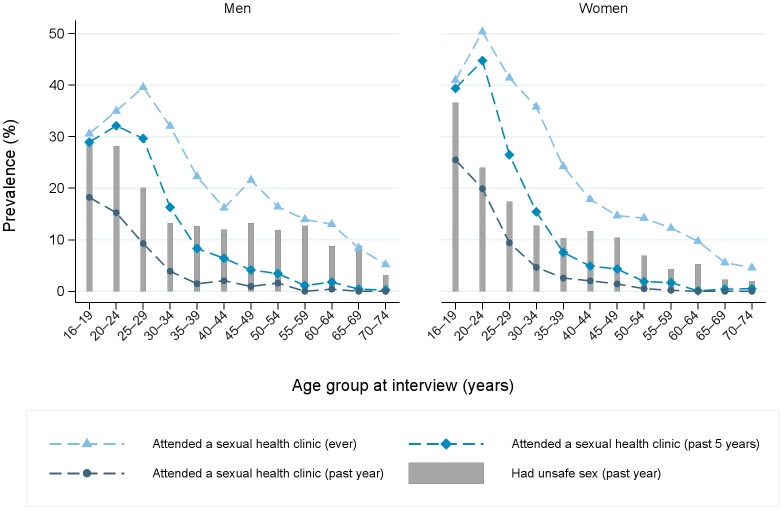
Prevalence of sexual health clinic attendance and unsafe sex (in last year) by sex and age group.

Online supplementary appendix table 1 gives the denominators for different subpopulations of the sample. In total, 5707 men and 8218 women were sexually experienced (see online supplementary appendix table 1) and just over one-fifth of these sexually experienced men (22.3%) and women (23.2%) aged 16–74 reported ever having attended a SHC, 12% having attended in the past 5 years and around 5% in the past year (see figure 1 and online supplementary appendix table 2). There was considerable variation by age, with attendance highest among young people: 19% of men and 26% of women aged 16–19 years had attended in the past year compared with 10% of those aged 25–29 and less than 1.5% of those aged 45 and over.

10.1136/sextrans-2017-053193.supp1 Supplementary file 1

### Patterns of SHC attendance among sexually active men and women aged 16–74 years

Among 4819 men and 6668 women aged 16–74 years who were sexually active (see online supplementary appendix 1), 4.9% (95%CI 4.3 to 5.4) of men and 6.0% (95%CI 5.5 to 6.6) of women reported attending a SHC in the past year (see online supplementary appendix table 3). After adjustment for age, those who attended SHCs in the past year were more likely to report a range of sexual behaviours considered to be markers of STI risk, including multiple sexual partners and concurrency (all past year) and were more likely to perceive themselves at risk of STIs.

### SHC non-attendance among those reporting unsafe sex in the past year

Approximately 15% of sexually active men and women aged 16–74 years had had unsafe sex in the past year (n=1002 and n=1253, respectively, see online supplementary appendix table 1). Of the men reporting unsafe sex, 59% (95% CI 56 to 63) reported not using a condom at first sex with a new vaginal or anal sex partner in the past year, 10% (95% CI 8 to 13) reported two or more sexual partners in the past year and no condom use in that time and a further 31% (95% CI 28 to 35) reported both of these markers of unsafe sex. Corresponding values in women were 63% (95% CI 60 to 66), 6% (95% CI 5 to 9) and 30% (95% CI 27 to 33).

Of those classified as having had unsafe sex, 89% of men and 82% of women reported *not* having attended a SHC in the past year (table 1). Non-attenders were more likely to be older and, among women, to report cohabiting with a partner (vs not being in a ‘steady’ relationship) and having no qualifications or only those typically gained at age 16. After adjustment for age, non-attenders tended to report fewer markers of risk, being less likely to report concurrent partnerships (among women), more than one sexual partner (among women) or more than two sexual partners (among men), one or more partnership with an age gap of 5 or more years (men), anal sex (women) and using the internet to find a sexual partner (all in the past year). Non-attenders were also less likely to have had one or more partner who normally lives outside the UK (past 5 years) and to perceive themselves greatly/quite a lot at risk of STIs.

**Table 1:** Comparison of sexual health (GUM) clinic attenders and non-attenders in the past year, among those (16–74 years) classified as having had unsafe sex (past year), by sex

	Men					Women				
	Attenders	Non-attenders	aOR for non-attendance*	95% CI	p Value†	Attenders	Non-attenders	aOR for non-attendance*	95% CI	p Value†
**Denominators (unwt, wt)**	***146, 110***	***856, 877***				***267, 149***	***986, 666***			
Sociodemographic factors										
Age at interview					<0.0001					<0.0001
16–24	64.4%	23.6%	1.00			62.4%	26.6%	1.00		
	(54.7% to 73.0%)	(20.9% to 26.6%)				(55.3% to 69.1%)	(23.8% to 29.6%)			
25–34	20.5%	21.4%	2.84	(1.76 to 4.58)		23.1%	24.6%	2.49	(1.71 to 3.63)	
	(14.5% to 28.3%)	(18.6% to 24.5%)				(17.7% to 29.6%)	(21.7% to 27.7%)			
35–44	7.9%	18.0%	6.23	(2.31 to 16.82)		10.1%	21.0%	4.86	(2.51 to 9.42)	
	(3.2% to 18.1%)	(14.8% to 21.7%)				(5.7% to 17.5%)	(17.9% to 24.5%)			
45+	7.2%	37.0%	13.95	(5.92 to 32.85)		4.3%	27.8%	15.22	(6.64 to 34.87)	
	(3.3% to 15.2%)	(33.2% to 41.0%)				(2.0% to 9.1%)	(24.3% to 31.7%)			
Relationship status					0.3864					0.0082
Married or civil partnership	3.7%	27.8%	3.68	(0.63 to 21.50)		5.1%	22.0%	2.23	(0.88 to 5.60)	
	(0.7% to 17.7%)	(24.0% to 31.9%)				(2.3% to 11.0%)	(18.6% to 25.8%)			
Living with partner of opposite or same sex	15.8%	14.4%	1.07	(0.58 to 1.97)		7.3%	16.3%	2.64	(1.31 to 5.33)	
	(9.9% to 24.3%)	(12.0% to 17.3%)				(4.0% to 12.9%)	(13.7% to 19.2%)			
In a ‘steady’ ongoing relationship but not living together	29.0%	24.0%	1.27	(0.82 to 1.97)		40.4%	25.5%	0.89	(0.63 to 1.27)	
	(21.8% to 37.3%)	(21.2% to 27.1%)				(33.9% to 47.2%)	(22.6% to 28.6%)			
Not in ‘steady’ relationship	51.5%	33.8%	1.00			47.2%	36.2%	1.00		
	(42.4% to 60.5%)	(30.3% to 37.4%)				(40.1% to 54.4%)	(32.8% to 39.8%)			
Academic qualifications**‡**					0.6424					0.0001
No academic qualifications	11.9%	22.6%	0.98	(0.50 to 1.91)		6.2%	19.6%	2.62	(1.32 to 5.19)	
	(7.4% to 18.7%)	(19.3% to 26.3%)				(3.5% to 11.0%)	(16.6% to 23.0%)			
Academic qualifications typically gained at age 16 years	39.1%	36.1%	0.81	(0.50 to 1.30)		23.0%	38.9%	2.09	(1.43 to 3.07)	
	(30.7% to 48.1%)	(32.5% to 40.0%)				(17.8% to 29.3%)	(35.3% to 42.7%)			
Studying for or have attained further academic qualifications	49.0%	41.2%	1.00			70.7%	41.4%	1.00		
	(39.7% to 58.4%)	(37.3% to 45.2%)				(63.8% to 76.8%)	(37.7% to 45.3%)			
Quintile of Index of Multiple Deprivation§					0.219					0.0028
1–2 (least deprived)	36.9%	34.4%	0.78	(0.50 to 1.24)		39.7%	32.8%	0.74	(0.50 to 1.12)	
	(28.5% to 46.2%)	(30.6% to 38.3%)				(32.0% to 47.9%)	(29.2% to 36.6%)			
3	14.9%	18.3%	1.29	(0.76 to 2.19)		11.6%	21.2%	1.77	(1.14 to 2.73)	
	(9.9% to 21.7%)	(15.4% to 21.7%)				(8.3% to 16.1%)	(18.2% to 24.6%)			
4–5 (most deprived)	48.2%	47.3%	1.00			48.7%	46.0%	1.00		
	(39.4% to 57.2%)	(43.3% to 51.4%)				(41.0% to 56.5%)	(42.1% to 49.9%)			
Sexual behaviours										
Had first heterosexual intercourse before 16 years¶					0.1168					0.191
No	51.1%	63.8%	1.00			55.2%	70.4%	1.00		
	(42.1% to 60.2%)	(60.1% to 67.4%)				(48.1% to 62.2%)	(67.1% to 73.6%)			
Yes	48.9%	36.2%	0.73	(0.49 to 1.08)		44.8%	29.6%	0.80	(0.57 to 1.12)	
	(39.8% to 57.9%)	(32.6% to 39.9%)				(37.8% to 51.9%)	(26.4% to 32.9%)			
Number of sexual partners, past year^**^					<0.0001					<0.0001
1	22.9%	38.4%	1.00			20.3%	49.3%	1.00		
	(16.0% to 31.7%)	(34.7% to 42.2%)				(15.3% to 26.5%)	(45.6% to 52.9%)			
2	12.4%	29.9%	1.43	(0.73 to 2.77)		26.9%	25.8%	0.45	(0.29 to 0.72)	
	(7.8% to 19.2%)	(26.4% to 33.7%)				(20.8% to 33.9%)	(22.7% to 29.3%)			
3–4	31.9%	19.6%	0.41	(0.23 to 0.74)		31.7%	15.4%	0.30	(0.19 to 0.46)	
	(23.7% to 41.4%)	(16.6% to 22.8%)				(25.8% to 38.1%)	(13.1% to 18.0%)			
5+	32.7%	12.1%	0.25	(0.14 to 0.44)		21.2%	9.5%	0.31	(0.18 to 0.54)	
	(24.8% to 41.7%)	(9.9% to 14.8%)				(15.7% to 28.0%)	(7.5% to 11.9%)			
Concurrency, past year					0.0891					<0.0001
No	61.9%	67.1%	1.00			58.1%	78.4%	1.00		
	(52.1% to 70.8%)	(62.8% to 71.1%)				(50.3% to 65.6%)	(74.9% to 81.5%)			
Yes	38.1%	32.9%	0.67	(0.42 to 1.06)		41.9%	21.6%	0.43	(0.29 to 0.64)	
	(29.2% to 47.9%)	(28.9% to 37.2%)				(34.4% to 49.7%)	(18.5% to 25.1%)			
At least one partnership with age gap of at least 5 years, past year					0.0049					0.256
No	55.8%	45.1%	1.00			55.2%	48.6%	1.00		
	(46.2% to 64.9%)	(41.2% to 49.1%)				(48.2% to 62.0%)	(45.0% to 52.3%)			
Yes	44.2%	54.9%	0.53	(0.34 to 0.83)		44.8%	51.4%	0.82	(0.58 to 1.15)	
	(35.1% to 53.8%)	(50.9% to 58.8%)				(38.0% to 51.8%)	(47.7% to 55.0%)			
Used the internet to find a sexual partner, past year					0.0001					0.0659
No	72.7%	84.9%	1.00			87.6%	90.9%	1.00		
	(64.6% to 79.5%)	(81.9% to 87.5%)				(80.4% to 92.4%)	(88.8% to 92.7%)			
Yes	27.3%	15.1%	0.39	(0.25 to 0.62)		12.4%	9.1%	0.52	(0.26 to 1.04)	
	(20.5% to 35.4%)	(12.5% to 18.1%)				(7.6% to 19.6%)	(7.3% to 11.2%)			
Anal sex with opposite sex partner, past year					0.2436					0.0053
No	67.4%	75.3%	1.00			66.9%	81.2%	1.00		
	(58.8% to 75.0%)	(71.8% to 78.5%)				(60.2% to 73.0%)	(78.2% to 83.8%)			
Yes	32.6%	24.7%	0.78	(0.52 to 1.18)		33.1%	18.8%	0.59	(0.41 to 0.85)	
	(25.0% to 41.2%)	(21.5% to 28.2%)				(27.0% to 39.8%)	(16.2% to 21.8%)			
Had 1+ same sex partner, past year										
No	93.3%	96.4%				91.6%	97.2%			
	(86.9% to 96.7%)	(94.6% to 97.7%)				(85.3% to 95.4%)	(95.9% to 98.1%)			
Yes	6.7%	3.6%	††			8.4%	2.8%	††		
	(3.3% to 13.1%)	(2.3% to 5.4%)				(4.6% to 14.7%)	(1.9% to 4.1%)			
Had 1+ partner who normally lives outside the UK, past 5 years					0.0371					0.0426
No	69.7%	81.0%	1.00			83.2%	90.2%	1.00		
	(60.0% to 77.9%)	(77.7% to 84.0%)				(76.6% to 88.3%)	(88.0% to 92.1%)			
Yes	30.3%	19.0%	0.59	(0.35 to 0.97)		16.8%	9.8%	0.57	(0.33 to 0.98)	
	(22.1% to 40.0%)	(16.0% to 22.3%)				(11.7% to 23.4%)	(7.9% to 12.0%)			
STI risk perception					0.0062					0.0048
Not at all at risk	29.7%	48.2%	1.00			37.5%	57.9%	1.00		
	(21.4% to 39.6%)	(44.2% to 52.1%)				(30.5% to 45.1%)	(54.2% to 61.5%)			
Not very much	46.6%	42.3%	0.80	(0.48 to 1.31)		44.7%	33.9%	0.65	(0.44 to 0.95)	
	(37.5% to 55.9%)	(38.6% to 46.1%)				(37.2% to 52.3%)	(30.5% to 37.5%)			
Greatly/Quite a lot at risk	23.7%	9.5%	0.41	(0.23 to 0.73)		17.9%	8.2%	0.43	(0.25 to 0.74)	
	(17.0% to 32.0%)	(7.6% to 11.8%)				(12.2% to 25.3%)	(6.4% to 10.3%)			
**Among 16–44 year olds denominators (unwt, wt)**	***139*, *102***	***621, 553***				***260, 142***	***811, 481***			
Reported testing for Chlamydia, past year										
No	10.3%	81.3%				13.9%	61.0%			
	(6.3% to 16.4%)	(77.7% to 84.4%)				(9.4% to 20.1%)	(57.0% to 64.8%)			
Yes	89.7%	18.7%				86.1%	39.0%			
	(83.6% to 93.7%)	(15.6% to 22.3%)				(79.9% to 90.6%)	(35.2% to 43.0%)			

*Age-adjusted ORs, adjusting for age as a continous variable.

†pValues for wald test for association between each variable and non-attendance after adjustment for age

‡Participants aged ≥17 years.

§Index of Multiple Deprivation is an area-level measure of deprivation [ref Payne & Abel].^33^

¶First heterosexual sex age 13+.

**Opposite and/or same-sex partners.

††ORs not presented due to cell sizes<30.

GUM, genitourinary medicine.

We examined chlamydia testing in the past year among 16–44 year olds reporting unsafe sex (table 1). Of those who had *not* attended a SHC in the past year, 18.7% (15.6–22.3) of men and 39.0% (35.2–43.0) of women had tested for chlamydia in that timeframe. Chlamydia testing was significantly more commonly reported among those aged <25 years: 27% of men and 59% of women of this age who had not attended a SHC had been tested vs 14% of men and 27% of women aged 25–44 years.

### Hypothetical service preferences for STI diagnosis/treatment among those reporting unsafe sex in the past year

Among 16–44 year olds reporting unsafe sex in the past year, around half reported that they would seek diagnosis and/or treatment from GP if they thought they might have an STI (50% of men and 54% of women, table 2). However, this varied substantially by whether they had previously attended a SHC. Among those who had never attended a SHC, 65.1% of men and 77.1% of women would seek treatment from GP. For those who had previously attended a SHC, SHCs were the most commonly preferred place of diagnosis and/or treatment, and preference was higher among those who had attended recently (75% of men and 77% of women who had attended a SHC in the past year). Few people would seek diagnosis and/or treatment from other locations.

**Table 2:** Preferred location to seek diagnosis or treatment for a suspected STI (hypothetical) among 16–44 year olds reporting unsafe sex within the past year, by sex and SHC attendance

	Men				Women			
	SHC attendance				SHC attendance			
	Within the last year	Attended but not within the last year	Never attended	Total	Within the last year	Attended but not within the last year	Never attended	Total
	15.6%	20.5%	63.9%	100.0%	22.8%	23.7%	53.4%	100.0%
	(13.1% to 18.5%)	(17.4% to 24.0%)	(59.7% to 67.8%)		(19.9% to 26.0%)	(21.0% to 26.7%)	(49.9% to 56.9%)	
Place would first go to seek diagnosis/treatment for a suspected STI								
General practice surgery	22.0%	26.0%	65.1%	50.4%	16.5%	38.7%	77.1%	54.1%
	(15.7% to 29.9%)	(19.3% to 34.1%)	(59.8% to 70.1%)	(46.1% to 54.6%)	(11.8% to 22.7%)	(32.3% to 45.6%)	(72.6% to 81.0%)	(50.6% to 57.6%)
Sexual health clinic (GUM clinic)	75.3%	70.3%	26.2%	42.9%	77.3%	55.2%	13.5%	38.0%
	(67.1% to 82.0%)	(62.1% to 77.4%)	(21.8% to 31.2%)	(38.7% to 47.2%)	(70.7% to 82.9%)	(48.4% to 61.9%)	(10.2% to 17.6%)	(34.5% to 41.6%)
Retail/other^*^	0.3%	1.5%	4.7%	3.3%	0.5%	1.1%	1.8%	1.3%
	(0.0% to 1.9%)	(0.5% to 4.8%)	(2.7% to 8.1%)	(2.0% to 5.6%)	(0.1% to 3.4%)	(0.3% to 3.5%)	(0.9% to 3.6%)	(0.7% to 2.4%)
Community clinic and other medical†	2.5%	2.1%	4.0%	3.4%	5.6%	4.9%	7.7%	6.6%
	(0.9% to 6.5%)	(0.8% to 5.7%)	(2.4% to 6.6%)	(2.2% to 5.1%)	(3.3% to 9.5%)	(2.7% to 8.9%)	(5.6% to 10.3%)	(5.1% to 8.4%)
Denominators								
Unweighted	139	167	454	760	260	266	546	1072
Weighted	102	134	418	655	142	148	333	623

*Retail includes: internet site offering treatment (1.3% men, 0.7% women), pharmacy/chemist (0.6% men, 0.2% women) as well as ‘somewhere else’ (1.5% men, 0.4% women).

†Community clinic and other medical includes: NHS FP clinic/contraceptive clinic/reproductive health clinic (2.0% men, 4.5% women), NHS antenatal clinic/midwife (0.4% men, 0.4% women), private non-NHS clinic or doctor (0.1% men, 0.8% women), youth advisory clinic, for example, Brook Clinic (0.3% men, 0.9% women) and hospital accident and emergency A&E department (0.6% men, 0.0% women).

GUM, genitourinary medicine, SHC, sexual health clinic.

## Discussion

Approximately 1 in 20 sexually active 16–74 year olds in Britain reported SHC attendance in the last year. Among those who we defined as having had unsafe sex in the past year, 89% of men and 82% of women had not attended a SHC during this timeframe. Clinic non-attenders were more likely to be older than those who had attended a clinic and were less likely to report other behaviours known to be associated with potential STI transmission risk, including greater numbers of sexual partners and concurrent partnerships. Additionally, many of those reporting unsafe sex, especially those aged <25 years and particularly women, who had not attended a SHC had been tested for chlamydia in the past year. However, there remain a large proportion of those reporting unsafe sex who had *neither* attended a SHC *nor* been tested for chlamydia in the past year indicating that further efforts are needed to reach those who may be at risk of STI acquisition or transmission. This tallies with other data from Natsal-3 which show that while overall, SHC attendees are at higher risk than non-attendees, many of those with prevalent chlamydia or reporting risk factors for chlamydia transmission had not recently tested.^9 19^

Much research on sexual health service use has been conducted within health service settings, omitting non-users of services. By using national probability sample survey data, we were able to estimate the proportion of the population not accessing SHCs. Furthermore, Natsal links population risk factors and service use behaviours, enabling us to assess the extent to which at-risk individuals access services. However, the cross-sectional nature of the study means that we cannot determine temporality; we do not know whether SHC attendance preceded or followed behaviours known to be potential indicators of need for sexual healthcare. The data presented are representative of individuals aged 16–74 years living in private residential households only; those under 16, the homeless and those living in institutions may have different profiles of SHC attendance and/or preferred sources for the diagnosis and/or treatment of STIs.

We also acknowledge limitations in our measures. First, our measure of SHC non-attendance does not take into account testing for STIs in other settings, such as primary care or community-based testing, which is particularly relevant for chlamydia and HIV as a substantial proportion of testing for these STIs occurs outside of SHCs.^20 21^ However, we have quantified chlamydia testing among those not attending SHC (for those aged <45 years on whom we have these data). Second, our two questions asking about SHC attendance and hypothetical service preference do not capture the increasing complexity of sexual health service provision in Britain. Given the broad range of settings providing SH services, we cannot be sure how participants interpreted *‘sexual health clinic (GUM clinic)*’; however, we obtained similar estimates of SHC attendance to those from GUM clinic surveillance data.^9^ Finally, we defined those with STI testing needs as participants reporting (in the past year) unprotected first sex with a new partner or more than one partner and no use of condoms. This refers to condom use for vaginal and anal sex since Natsal-3 did not ask about condom use during oral sex, which is a limitation as transmission risk for many STIs is determined by condomless *oral* sex. In addition, we acknowledge that an individual’s STI risk extends beyond their own behaviour as it also reflects the characteristics and behaviours of their partner(s) and wider sexual networks. This means our measure will overestimate risk for some and underestimate risk for others. In particular, while we recognise that risks may differ by age and age of sexual partners, we did not have the power to stratify analysis by age. Nonetheless, this measure does provide some indicator of exposure to risk and is in line with national clinical recommendations for those who have had unprotected sex with a new partner to have a sexual health check.^17^

In terms of the extent of unmet sexual health need in the population, we found that over 85% of men and women who we considered to have had unsafe sex had not attended a SHC in the past year. While this suggests there is unmet need, the exact intensity of resource required to meet this need, that is, a comprehensive range of STI tests available from specialist GUM services or a chlamydia test accessed online or through GP, cannot be determined from these data and the take home message is less pessimistic.

First, those attending clinic were younger and were more likely to report higher-risk sexual behaviours. These are individuals who may not receive comprehensive sexual healthcare in primary care settings, when compared with a SHC, where the full range of STI tests, appropriate treatment, provider-led partner notification and health promotion support are more likely to be available,^22–24^ and it is encouraging that SHC use is higher among these key populations.

Second, many of those reporting unsafe sex and *not* attending a SHC reported testing for chlamydia in the past year, suggesting engagement with sexual health services more broadly. Our previous work suggested that most of those who test for chlamydia outside SHCs were at lower risk^25^ and may therefore not need the more specialist services provided by a SHC. The majority of people reporting unsafe sex would seek diagnosis and treatment for a suspected STI from GP, demonstrating the potential for sexual healthcare provision in primary care to reach those who do not attend specialist services and do not need to, particularly given that the majority of the British population are registered with a GP. Testing within GP may be increasingly feasible with current reconfigurations and retendering of SHCs across England and the drive to achieve more cost-effective ways of testing and treating people. Strong referral systems between local healthcare providers are required to ensure that those requiring more specialist services access them.

SHCs remain stigmatised services but qualitative research^26^ shows attendance helps normalise the experience and reduces felt stigma and the fear of discrimination.^27^ Those who had more recently attended a SHC were more likely to report that they would seek care for a suspected STI at a SHC. This might be considered a marker of patient satisfaction provided a choice of services is available. At the same time, our data suggest that SHC providers should educate people about what to expect when they attend a clinic^28^ and normalise attendance to increase familiarity with SHCs. This is particularly important for those who engage in high-risk behaviours for STI/HIV transmission. The data do not allow us to investigate why people chose particular services and further research is required to draw conclusions about this and gauge satisfaction with particular sexual health services.

Although <2% of men and <1% of women would first seek diagnosis and/or treatment for a suspected STI from an internet site offering treatment, these data were collected in 2010–2012 and online sexual health services have expanded and continue to do so in Britain as in other countries.^29 30^ eSexual Health services are likely to become more widely used. These may act as a triage, referring those at higher risk or with a positive test result. Such services offering diagnosis and/or treatment have been found to be highly acceptable to users^31^ and may address some of the barriers to SHC attendance.^32^

Many of the current initiatives to improve sexual health in Britain focus on young people, a group at highest risk of some sexual health outcomes such as chlamydia infection.^9 19^ Young people (16–24 years) were more likely to report SHC attendance (ever), despite having fewer years of being sexually active, which suggests that efforts to promote sexual health and service use in this group have had some success. Attendance at SHC was also higher in younger than older people reporting unsafe sex and in addition many young people, particularly women, who had not attended a SHC reported chlamydia testing. Although behaviours known to be associated with STI transmission, such as condomless sex with multiple partners, are more common among young people, they are not infrequently reported by men and women aged 25–44^1^, and therefore sexual health and advice services should include those open to older adults/targeted by risk behaviour rather than age, although not at the expense of services targeting young people.

People reporting behaviours that are markers of potential STI transmission risk were more likely to report recent SHC attendance and many of those not attending SHCs reported testing for chlamydia. While it is encouraging that large and increasing proportions of the population reporting sexual behaviours known to be associated with STI transmission are accessing services, many people still do not do so. Given that non-attenders reporting risk behaviours expressed preference for GP care, this may be an avenue for improving access to STI testing. However, this must be alongside improved referral from GP to more specialist services for patients with more complex needs to ensure they receive a more comprehensive package of care, including partner notification.

**Key messages**

- Approximately 1 in 20 sexually active 16–74 year olds in Britain reported sexual health clinic (SHC) attendance in the last year.
- However, more than 85% of British people aged 16–74 years reporting ‘unsafe sex’ in the past year had not attended a SHC in that period.
- Although among those reporting unsafe sex, attendance was higher in those reporting more sexual partners and concurrency, both in the past year and many non-attenders reported testing for chlamydia in the past year.
- Diverse sexual health services with strong referral mechanisms are needed to offer appropriate care to those at risk.
