# Supplementary material for: Sexual health clinic attendance and non-attendance in Britain: findings from the third National Survey of Sexual Attitudes and Lifestyles (Natsal-3)
Source: Sex Transm Infect. 2017 Sep 29;94(4):268–76. doi: 10.1136/sextrans-2017-053193 (PMC5969324; doi:10.1136/sextrans-2017-053193)
Supplement: Supplementary file 1 [file sextrans-2017-053193supp001.pdf]

Web Appendix Table 1: Denominators for each subpopulation of the Natsal-3 sample by age and sex

		Men							Women						
		16-24	25-34	35-44	45-54	55-64	65-74	Total	16-24	25-34	35-44	45-54	55-64	65-74	Total
All participants	Unwt	1729	1525	806	794	775	667	6293	2140	2487	1215	1123	1030	874	8869
	Wt	1238	1374	1425	1413	1197	860	7508	1207	1380	1455	1443	1235	935	7654
Sexually experienced [2]	*% (of row above)* [1]	81.0%	94.9%	97.9%	96.3%	93.6%	91.6%	92.9%	80.2%	95.9%	97.9%	97.4%	96.1%	93.4%	93.8%
	Unwt	1375	1456	790	761	715	610	5707	1740	2397	1189	1091	985	816	8218
	Wt	1003	1304	1395	1360	1121	788	6971	968	1323	1425	1406	1189	873	7181
Data on SHC attendance (ever)	*% (of row above)* [1]	99.9%	99.5%	98.9%	98.9%	97.9%	97.0%	98.8%	99.6%	99.3%	98.6%	97.9%	99.1%	97.3%	98.7%
	Unwt	1373	1448	783	751	699	586	5640	1731	2378	1175	1066	976	794	8120
	Wt	1003	1304	1395	1360	1121	788	6971	968	1313	1405	1376	1176	850	7084
Data on time since last SHC attendance	*% (of row above)* [1]	98.6%	97.8%	99.1%	99.3%	99.6%	99.8%	99.0%	98.3%	99.5%	99.5%	99.6%	99.5%	99.7%	99.4%
	Unwt	1353	1418	775	745	696	585	5572	1701	2365	1168	1060	970	791	8055
	Wt	987	1269	1368	1336	1093	762	6814	947	1306	1398	1371	1170	847	7039
Sexually active [3]	*% (of row above)* [1]	94.6%	95.5%	94.6%	89.5%	78.5%	61.5%	87.5%	96.7%	95.3%	92.8%	87.0%	65.0%	42.5%	82.0%
	Unwt	1277	1350	717	635	515	325	4819	1650	2232	1056	872	573	285	6668
	Wt	934	1212	1294	1196	858	469	5962	916	1244	1297	1192	760	360	5770
Unsafe sex [4]	*% (of row above)* [1]	29.8%	17.3%	12.9%	13.8%	13.7%	10.8%	16.6%	29.5%	15.9%	11.9%	10.2%	7.1%	4.5%	14.1%
	Unwt	388	258	114	113	93	36	1002	515	401	155	117	52	13	1253
	Wt	278	210	167	164	119	50	987	270	198	155	122	54	16	815

[1] % are of weighted denominators

[2] 1+ lifetime partner (opposite and/or same-sex)

[3] 1+ partner (opposite and/or same-sex) in the past year

[4] Defined as either not using a condom at first sex with a new (vaginal or anal sex) partner in the oast year and/or two or more sexual partners in the past year and no condom use in that time

Web Appendix Table 2: Prevalence of sexual health (GUM) clinic attendance in sexually experienced by age and sex

	16-19 years	20-24 years	25-29 years	30-34 years	35-39 years	40-44 years	45-49 years	50-54 years	55-59 years	60-64 years	65-69 years	70-74 years	All ages (16-74)
**Men**													
**Ever**	30.7%	35.0%	39.7%	32.3%	22.7%	16.2%	21.8%	16.6%	14.1%	13.4%	8.6%	5.4%	22.3%
	[26.4%,35.3%]	[31.4%,38.7%]	[35.9%,43.6%]	[28.4%,36.5%]	[18.6%,27.4%]	[12.8%,20.4%]	[17.6%,26.6%]	[12.9%,21.1%]	[10.3%,19.1%]	[10.2%,17.4%]	[6.0%,12.2%]	[3.1%,9.2%]	[21.1%,23.6%]
**Past 5 years**	29.5%	32.6%	30.3%	16.9%	8.5%	6.5%	4.2%	3.5%	1.1%	1.8%	0.4%	0.3%	11.5%
	[25.2%,34.2%]	[29.1%,36.3%]	[26.8%,34.0%]	[14.0%,20.2%]	[6.1%,11.8%]	[4.3%,9.7%]	[2.6%,6.8%]	[2.0%,6.0%]	[0.4%,3.2%]	[0.9%,3.6%]	[0.1%,1.8%]	[0.0%,1.9%]	[10.7%,12.4%]
**Past year**	18.6%	15.5%	9.5%	4.0%	1.5%	2.1%	1.0%	1.6%	0.0%	0.4%	0.0%	0.0%	4.3%
	[15.3%,22.4%]	[12.9%,18.5%]	[7.6%,11.7%]	[2.7%,6.1%]	[0.7%,3.1%]	[1.0%,4.1%]	[0.4%,2.5%]	[0.7%,3.6%]		[0.1%,1.3%]			[3.9%,4.8%]
**Women**													
**Ever**	41.2%	50.5%	41.6%	36.1%	24.6%	18.0%	14.9%	14.6%	12.4%	9.8%	5.6%	4.8%	23.2%
	[37.2%,45.3%]	[47.2%,53.9%]	[38.6%,44.7%]	[32.9%,39.4%]	[21.2%,28.5%]	[15.1%,21.4%]	[12.1%,18.2%]	[11.6%,18.3%]	[9.6%,15.9%]	[7.4%,12.9%]	[3.8%,8.4%]	[2.9%,7.8%]	[22.2%,24.2%]
**Past 5 years**	40.1%	45.8%	26.8%	15.7%	7.7%	5.0%	4.4%	2.0%	1.7%	0.1%	0.4%	0.5%	11.9%
	[36.1%,44.2%]	[42.5%,49.3%]	[23.9%,29.8%]	[13.3%,18.4%]	[5.7%,10.3%]	[3.5%,7.0%]	[3.0%,6.3%]	[1.0%,3.6%]	[0.9%,3.3%]	[0.0%,0.7%]	[0.1%,1.4%]	[0.1%,2.1%]	[11.2%,12.7%]
**Past year**	26.0%	20.4%	9.5%	4.7%	2.6%	2.1%	1.4%	0.5%	0.2%	0.0%	0.0%	0.0%	5.0%
	[22.5%,29.7%]	[17.6%,23.5%]	[7.8%,11.6%]	[3.4%,6.6%]	[1.5%,4.7%]	[1.2%,3.6%]	[0.7%,2.8%]	[0.2%,1.5%]	[0.0%,1.4%]				[4.6%,5.5%]
**Denominators**													
**Men**													
Unweighted	*582*	*793*	*826*	*630*	*382*	*408*	*401*	*360*	*335*	*380*	*354*	*256*	*5707*
Weighted	*374*	*629*	*651*	*654*	*671*	*724*	*716*	*644*	*540*	*581*	*474*	*313*	*6971*
**Women**													
Unweighted	*675*	*1065*	*1364*	*1033*	*591*	*598*	*589*	*502*	*474*	*511*	*482*	*334*	*8218*
Weighted	*344*	*624*	*670*	*653*	*673*	*751*	*762*	*644*	*597*	*589*	*541*	*333*	*7181*

Web Appendix Table 3: Sexual health (GUM) clinic attendance by sociodemographic and behavioural characteristics among sexually active 16-74 year olds, by sex

	Attended a sexual health clinic in the past year											
	Men						Women					
	%	95% CI	aOR[1]	95% CI	p value	Denominators (unwt, wt)	%	95% CI	aOR[1]	95% CI	p value	Denominators (unwt, wt)
**Total**	4.9%	(4.3-5.4)				*4819, 5962*	6.0%	(5.5-6.6)				*6668, 5770*
**Sociodemographic factors**												
**Age at interview**					<0.0001						<0.0001	
16-19	19.6%	(16.1-23.6)	1	-		*533, 344*	26.7%	(23.2-30.5)	1	-		*644, 329*
20-24	16.0%	(13.3-19.1)	0.78	(0.57-1.08)		*744, 590*	21.0%	(18.1-24.2)	0.73	(0.57-0.95)		*1006, 587*
25-34	7.0%	(5.8-8.5)	0.31	(0.23-0.43)		*1350, 1212*	7.4%	(6.2-8.8)	0.22	(0.17-0.28)		*2232, 1244*
35-44	1.8%	(1.1-3.1)	0.08	(0.04-0.14)		*717, 1294*	2.3%	(1.5-3.5)	0.07	(0.04-0.10)		*1056, 1297*
45-54	1.4%	(0.7-2.5)	0.06	(0.03-0.11)		*635, 1196*	1.1%	(0.6-2.0)	0.03	(0.02-0.06)		*872, 1192*
55-64	0.3%	(0.1-0.9)	0.01	(<0.01-0.04)		*515, 858*	0.2%	(0.0-1.1)	<0.01	(<0.01-0.03)		*573, 760*
65-74	0.0%	-	-	-		*325, 469*	0.0%	-	-	-		*285, 360*
**Region**					0.1161						<0.0001	
Rest of England	4.9%	(4.3-5.5)	1	-		*3680, 4356*	5.5%	(4.9-6.2)	1	-		*5015, 4226*
Greater London	6.1%	(4.3-8.6)	1.15	(0.77-1.73)		*480, 775*	11.0%	(8.9-13.6)	2.16	(1.60-2.90)		*694, 716*
Scotland	4.0%	(2.8-5.7)	0.81	(0.53-1.23)		*405, 537*	3.9%	(2.7-5.6)	0.66	(0.43-1.02)		*580, 533*
Wales	2.7%	(1.5-4.9)	0.5	(0.26-0.96)		*254, 295*	4.7%	(3.2-6.8)	0.81	(0.53-1.24)		*379, 294*
**Relationship status**					<0.0001						<0.0001	
Married or civil partnership	0.5%	(0.3-1.0)	1	-		*1864, 3269*	1.1%	(0.8-1.6)	1	-		*2713, 3196*
Living with partner of opposite or same sex	5.4%	(4.0-7.3)	5.42	(2.67-11.00)		*833, 990*	5.9%	(4.6-7.6)	2.07	(1.33-3.21)		*1266, 992*
In a 'steady' ongoing relationship but not living together	10.9%	(9.0-13.2)	8.02	(4.00-16.07)		*941, 755*	16.1%	(14.1-18.3)	4.55	(3.02-6.86)		*1348, 784*
Not in a 'steady' relationship	14.3%	(12.3-16.6)	11.2	(5.65-22.24)		*1177, 944*	15.8%	(13.6-18.4)	5.1	(3.38-7.71)		*1334, 793*
**Academic qualifications [2]**					0.4002						0.1214	
No academic qualifications	2.9%	(2.0-4.2)	1	-		*714, 987*	2.6%	(1.8-3.6)	1	-		*898, 900*
Academic qualifications typically gained at age 16 years	4.5%	(3.7-5.5)	0.81	(0.51-1.27)		*1630, 2011*	4.1%	(3.5-4.9)	0.88	(0.59-1.33)		*2307, 2030*
Studying for or have attained further academic qualifications	5.5%	(4.7-6.4)	0.73	(0.47-1.16)		*2281, 2791*	8.0%	(7.1-9.1)	1.13	(0.77-1.67)		*3171, 2632*
**Quintile of Index of Multiple Deprivation [3]**					0.6523						0.0031	
1 - 2 (least deprived)	4.0%	(3.2-4.8)	1	-		*1929, 2532*	4.9%	(4.1-5.8)	1	-		*2538, 2419*
3	4.5%	(3.5-5.9)	0.98	(0.69-1.39)		*936, 1166*	4.6%	(3.7-5.7)	0.73	(0.54-1.00)		*1299, 1118*
4 - 5 (most deprived)	6.0%	(5.1-7.1)	1.12	(0.84-1.48)		*1954, 2264*	7.9%	(6.9-9.1)	1.21	(0.94-1.55)		*2831, 2233*
**Sexual Behaviours**												
**Had first heterosexual intercourse before 16 years [4]**					<0.0001						0.03	
No	3.5%	(3.0-4.2)	1	-		*3345, 4318*	4.7%	(4.2-5.4)	1	-		*4908, 4571*
Yes	8.4%	(7.1-9.9)	1.86	(1.44-2.41)		*1425, 1579*	11.2%	(9.7-12.9)	1.28	(1.02-1.60)		*1692, 1143*
**Number of sexual partners, past year [5]**					<0.0001						<0.0001	
1	2.4%	(1.9-2.8)	1	-		*3545, 4796*	3.1%	(2.7-3.5)	1	-		*5424, 5006*
2	8.2%	(6.0-11.1)	1.92	(1.27-2.91)		*531, 504*	21.4%	(17.9-25.5)	4.47	(3.30-6.05)		*568, 364*
3-4	18.0%	(14.2-22.4)	4.74	(3.26-6.89)		*408, 360*	29.0%	(24.3-34.3)	5.37	(3.89-7.42)		*408, 227*
5+	26.7%	(21.4-32.7)	8.19	(5.60-11.97)		*296, 256*	35.4%	(27.4-44.3)	6.47	(4.14-10.12)		*221, 131*
**Concurrency, past year**					<0.0001						<0.0001	
No	3.7%	(3.2-4.3)	1	-		*3944, 5129*	4.1%	(3.7-4.6)	1	-		*5787, 5214*
Yes	14.9%	(11.7-18.8)	2.86	(2.01-4.07)		*452, 441*	31.5%	(26.5-37.0)	5.57	(4.03-7.69)		*411, 245*
**Had unsafe sex, past year**					<0.0001						<0.0001	
No	3.6%	(3.1-4.1)	1	-		*3771, 4920*	4.0%	(3.5-4.5)	1	-		*5362, 4905*
Yes	11.1%	(9.3-13.3)	2.26	(1.72-2.97)		*1002, 987*	18.3%	(15.9-20.9)	3.04	(2.37-3.89)		*1253, 815*
**Used the internet to find a sexual partner, past year**					<0.0001						<0.0001	
No	4.1%	(3.6-4.6)	1	-		*4472, 5645*	5.7%	(5.1-6.2)	1	-		*6448, 5622*
Yes	18.3%	(14.3-23.0)	4.37	(3.08-6.21)		*346, 315*	19.6%	(13.5-27.5)	4.4	(2.44-7.93)		*218, 146*
**Had 1+ same sex partner, past year**					<0.0001						0.0016	
No	4.4%	(3.9-5.0)	1	-		*4660, 5803*	5.7%	(5.2-6.3)	1	-		*6455, 5613*
Yes	20.8%	(14.6-28.8)	6.52	(3.76-11.33)		*159, 159*	16.3%	(11.3-22.9)	2.41	(1.40-4.16)		*213, 157*
**Ever experienced non-volitional sex**					0.0001						0.0046	
No	4.6%	(4.1-5.2)	1	-		*4685, 5809*	5.7%	(5.1-6.3)	1	-		*5813, 5071*
Yes	14.8%	(8.4-24.6)	4.37	(2.05-9.30)		*69, 81*	7.6%	(5.8-9.9)	1.63	(1.16-2.30)		*681, 575*
**STI risk perception**					<0.0001						<0.0001	
Not at all at risk	2.8%	(2.4-3.4)	1	-		*3147, 4294*	3.8%	(3.3-4.3)	1	-		*4906, 4530*
Not very much	8.1%	(6.8-9.6)	1.57	(1.17-2.10)		*1390, 1420*	13.0%	(11.2-15.0)	2.02	(1.59-2.58)		*1496, 1052*
Greatly / Quite a lot at risk	22.7%	(17.6-28.7)	4.83	(3.23-7.22)		*261, 224*	23.3%	(17.7-29.9)	3.78	(2.56-5.58)		*233, 161*

P-values are for the association between a variable and attendance at sexual health clinic after adjustment for age and were obtained using a Wald Test
Percentages are based on weighted denominators excluding missing data.

Footnotes:

[1] Age-adjusted ORs

[2] Participants aged ≥17 years

[3] Index of Multiple Deprivation is an area-level measure of deprivation [20].

[4] First heterosexual sex age 13+

[5] Opposite and/or same-sex partners
